# Shared decision making embedded in the undergraduate medical curriculum: A scoping review

**DOI:** 10.1371/journal.pone.0207012

**Published:** 2018-11-14

**Authors:** Marie-Anne Durand, Peter R. DiMilia, Julia Song, Renata W. Yen, Paul J. Barr

**Affiliations:** The Dartmouth Institute for Health Policy & Clinical Practice, Dartmouth College, Lebanon, United States of America; University of Birmingham, UNITED KINGDOM

## Abstract

**Objective:**

Shared decision making (SDM) training is shown to be effective and is increasingly embedded in continuing medical education. There is little evidence, however, about undergraduate medical education for SDM. The aim of this scoping review was to identify existing SDM training embedded in the undergraduate medical curriculum and analyze their impact.

**Methods:**

The authors systematically searched the extant literature for peer reviewed articles, hand searched key journals and reference lists of key articles, and contacted relevant stakeholders as part of a key informant analysis.

**Results:**

Included in the qualitative synthesis were 12 studies evaluating 11 SDM courses in medical education across six countries. Most courses integrated SDM training in clinical clerkship and varied in length from one to seven hours. The majority of studies assessed course impact on students’ skills in SDM. Most studies suggested that students’ skills and confidence in SDM significantly increased post-training, but three studies reported no significant improvement in SDM. Ten courses continue to be taught routinely.

**Conclusion:**

Overall, studies suggested a positive impact on medical students’ skills, confidence, and attitudes regarding SDM. Embedding SDM training in undergraduate medical education may be a practical and effective solution for current barriers to the widespread adoption of SDM.

## Introduction

The landscape of medicine is constantly evolving. One such movement in medicine has been towards engaging patients in their care through shared decision making (SDM).[1, 2] SDM encourages care teams and patients to discuss reasonable healthcare options together, using the best available evidence, so patients are supported to construct informed preferences about available options.[3] Patient decision aids may also be used to promote SDM. These interventions provide evidence-based information about the harms and benefits of reasonable healthcare options to help individuals deliberate about their preferences. Over the past decade, SDM and related interventions (e.g., patient decision aids) have demonstrated effectiveness in controlled contexts and garnered policy support worldwide.[4, 5] In the United States, the Patient Protection and Affordable Care Act encourages health organizations and healthcare professionals to promote patient engagement in health care and provide accessible, evidence-based information about options’ harms, benefits, and outcome probabilities.[2, 6] While the concept was introduced several decades ago,[7] implementation of SDM into clinical practice has been slow and difficult worldwide.[8, 9]

Various barriers to the implementation of SDM have been identified.[9–14] Time constraints, clinicians’ attitudes, and lack of understanding about the relevance and applicability of SDM are major obstacles to widespread adoption.[12, 15] Elwyn et al. described health professionals’ indifference to patient decision aids and associated organizational inertia.[12] Unless clinicians fully understand the principles and benefits of SDM and are trained in engaging patients in their care, widespread adoption of SDM is unlikely. Research suggests that successful implementation of SDM into routine care will require interventions targeting the clinicians, the patients, or, ideally, both.[16] SDM training appears to be effective in addressing common barriers to its implementation.[17] It is increasingly embedded in continuing medical education curriculum.[18, 19] As far as can be determined, SDM principles are not routinely taught in medical school curricula. There is little evidence as to which strategies are most effective for instructing medical school students about SDM.

The aim of this scoping review was to identify and analyze the literature on existing medical education that integrates SDM training into the undergraduate medical school curriculum.

## Methods

We conducted a scoping review using Arksey and O’Malley’s framework.[20] The scoping review methodology is recommended when the field of interest is complex and has not been comprehensively reviewed.[20] This approach was chosen to fully examine the extent and nature of research activity surrounding the evaluation of SDM training embedded in undergraduate medical school curricula. The findings will in turn be summarized and disseminated to facilitate and promote the integration of SDM into undergraduate medical school curricula.

Arksey and O’Malley's rigorous framework comprises the following stages: (1) Identifying the research questions; (2a) Identifying relevant studies; (2b) Consultation exercise; (3) Study selection; (4) Charting the data; (5) Collating, summarizing, and reporting the results.

### Stage 1. Identifying the research questions

Two research questions guided this scoping review:

1. What are the characteristics of medical training courses that integrate SDM into the undergraduate medical curriculum?
2. What is the impact of medical education that integrates SDM training into the undergraduate medical curriculum?

### Stage 2. Identifying relevant literature

#### Search strategy

We searched MEDLINE (PubMed) and Web of Science from their respective inceptions until June 2017 and EMBASE until April 2016 (end of institutional access) (see search strategy in supplemental file). The following search themes were combined: Medical education, shared decision making, curriculum and measurement (see Table 1). We hand searched key journals (Patient Education and Counseling, Medical Decision Making, Health Expectations, JAMA, and BMJ) to identify any relevant articles. We also searched ‘cited by’ and ‘related searches’ in PubMed as well as the reference lists of all included primary and review articles.

**Table 1:** Search terms^a^.

Themes	Search terms
Medical Education	Education, Medical
	Models, Educational
	Medical student
Shared decision making	Decision Making
	Patient Participation
	Physician-Patient Relations
	Shared decision making [keyword]
Curriculum	Curriculum Curriculum development
Measurement	Educational Measurement
Irrelevant	Education, Medical, Continuing
	General Surgery
	Career Choice
	Vocational Guidance

^a^Within each theme, search terms (MeSH and keywords) were combined using the Boolean operator “OR,” the operator “AND” was used to find the intersection of these themes, and the operator “NOT” was used to exclude consistently encountered “irrelevant” articles.

#### Consultation exercise

In addition to the search strategies outlined above, we consulted experts in the field to identify other unpublished research that would have evaluated, or is currently evaluating, the impact of training courses that integrate SDM into the undergraduate medical curriculum.

Key stakeholders were identified through discussions among the research team. We used existing contacts, an e-mail listserv (shared-l@shared-l.org, 579 members), and relevant social media pages (Shared@Facebook closed group, 708 members).

### Stage 3. Study selection

A set of inclusion and exclusion criteria were developed *a priori* to identify all studies that evaluated educational courses integrating SDM training of medical students into the curriculum. Two researchers (M-AD and PRD) independently screened all articles and abstracts for eligibility via electronic and manual searches. Disagreements about study inclusion were resolved by discussion with a third researcher (PJB).

Studies were included if the course: (1) was targeted at undergraduate allopathic medical education, 2) was integrated into the curriculum, 3) had been evaluated, irrespective of study type, 4) focused on SDM. Foreign language studies were excluded because of the cost and time involved in translating them into English.

For the purpose of the scoping review, we used the 2017 Cochrane systematic review definition of SDM: Shared decision making is defined as a process through which clinicians and patients make healthcare choices together, representing the crux of people centred care.[4, 21] SDM training courses (lasting 60 minutes minimum) embedded in communication skill programs were included in this review as long as they met the above criteria.

### Stage 4. Charting the data

Prior to beginning the review process, all authors developed, reviewed, and approved a standard protocol and search strategy. The research team subsequently developed a data-charting form. Two researchers (M-AD and PRD) piloted this form by independently extracting data on two studies and comparing their results. From this pilot, they added a column to capture information related to routine adoption of the SDM training course. No other changes were made. Using this form, two researchers (M-AD and PRD) independently extracted data on the remaining studies, including the study's country, type, purpose, and methodological approach. Data collected on the study methodology included sample size, inclusion and exclusion criteria, type of training course, medical school year, course duration, outcome measures and results, and continued integration in the medical curriculum. All study authors were contacted via email to ask any clarifying questions. If authors did not respond, they received a reminder one week and, if needed, two weeks after the original email.

M-AD and PRD resolved differences in data extraction by reaching consensus through discussion. If they could not reach consensus, they consulted a third member of the research team (PJB). In scoping reviews, assessing the quality of included studies is not part of the study remit.[20] We did not conduct a quality assessment.

### Stage 5. Collating, summarizing and reporting the results

Three members of the research team (JS, RWY, M-AD) tabulated and summarized the extracted data. A descriptive narrative has been used to present the findings.

## Results

**Fig 1:**
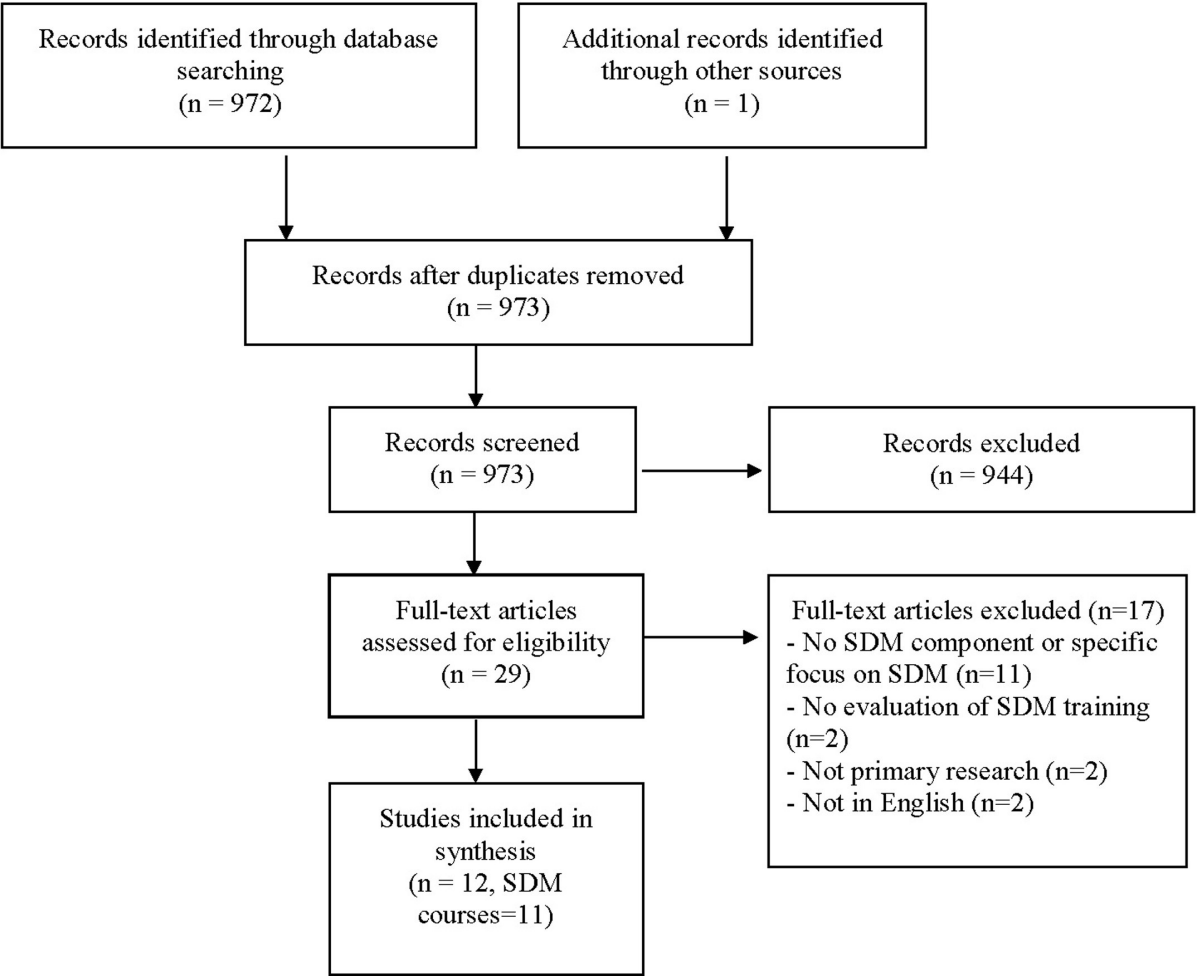
Study selection flow chart.

The database search, key journal search, and key informant analysis yielded a total of 973 results. The title/abstract screening resulted in 29 full-text articles for review. A final set of 12 articles met our inclusion criteria (Fig 1). Ten out of 11 authors contacted via email replied and provided additional information about the study and training course.

### Study characteristics

The scoping review included 12 studies which described 11 SDM training courses embedded in undergraduate medical education (see Table 2).[22–33] The studies were conducted in six countries: Australia (n = 1), the Netherlands (n = 1), Canada (n = 1), Switzerland (n = 1), Germany (n = 3), and the United States (n = 5). The study designs included one randomized controlled trial, six quasi-experimental studies, and five observational studies. Most studies have been published within the last 10 years (n = 9). Some training courses were interdisciplinary in nature and included students from other disciplines such as pharmacy and dentistry.[22, 30] For the purpose of this review, we only reported data (in text and table) related to undergraduate medical students. The total number of participants across all studies (medical students with complete data only) was 1,675 (ranging from 21 to 456 per study).

**Table 2:** Characteristics of included studies.

Author, year	Country	Study design	Participants	Duration and description of SDM training course	Outcomes	Routinely taught?
**Hoffmann, 2014**	Australia	Randomized control trial	76 3rd year medical students from two universities.	One-hour tutorial comprising a) introduction to a five-step framework for SDM, b) DVD of a modelled role-play demonstrating SDM skills c) facilitated critique of the role-play and group discussion about strategies to facilitate SDM. **Mandatory course**.	Intervention group improved SDM skills (19% improvement on OPTION scale), confidence in facilitating SDM (13% improvement on an 11-item confidence scale), and attitudes towards SDM (3% improvement on the sharing subscale of Patient Practitioner Orientation Scale (PPOS)).	Yes
**Hulsman, 2004**	The Netherlands	Observational	110 3rd year medical students.	Two 2-hour training sessions (4 hours) on decision making that included SDM. The 2-hour lessons started with a 50-minute introduction, followed by three roleplays and feedback of about 25 minutes each. **Mandatory course**.	The AMC Communication Test (AMC) presented films on history making, breaking bad news, and SDM, each followed by short essay questions. Students scored relatively poorly on SDM compared to history taking and breaking bad news.	Yes
**Kiessling, 2013**	Switzerland	Quasi-experimental	75 3rd year medical students.	1 hour of lecture and 2.5 hours of small group role-plays (3.5 hours) with pre-defined scripts. Emphasis was placed on patient-centered communication and the techniques aimed to give the patient a voice in the conversation and promote SDM. **Mandatory course**.	A questionnaire was used. Students felt more confident in all skills taught in the course including dealing with the patient's emotions, gathering the relevant information from the patient, and understanding what the patient was telling.	Yes
**Luttenberger, 2014**	Germany	Quasi-experimental	173 2nd year medical students.	The 1.5 hour SDM module was included in the “Physician-patient communication" course. The theoretical framework was presented in a lecture format combining SDM and motivational interviewing. SDM was practiced with two role plays. **Mandatory course**.	Students reported gaining competence in managing difficult communication situations with patients (82.1%) and considered having learned something useful for their role as a doctor (77.3%).	Yes
**Marko, 2015**	USA	Quasi-experimental	77 3rd year medical students.	2-hour training session including didactic review and role-play. The shared decision-making model was presented in the context of treatment decisions regarding miscarriage. Formative feedback was provided by faculty during the role-playing session. SDM content included taking history and focused discussion regarding patients' preferences for three available treatment options. **Mandatory course.**	A performance checklist that incorporated elements of shared decision-making was used pre- and post-training. At the end of the course, the intervention group showed a significant improvement compared to the control group (94.2% compared with 69.7%, p < .001).	Yes
**Morrow, 2011**	USA	Observational	73 3rd year medical students.	7.5 hours of experiential, small-group, and online learning designed to provide students with opportunities to learn content, practice skills, and share observations from their preceptorship about SDM. **Mandatory course.**	Students identified the most useful aspects of the course: (1) defining SDM, (2) learning how to use patient decision aids, and (3) viewing selected segments of their own and other students’ Simulated Patient Experience (SPE) videos.	Yes (curriculum modified after article publication)
**Mortsiefer, 2012 and 2014**	Germany	Observational	453 4th year medical students.	2 hours and 45-minute course divided into e-learning content (45 min) and role play using standardized patients (2 hours). The SDM course was embedded in the CoMeD program (Communication in Medical Education Dusseldorf) aimed at teaching challenging conversational issues. The SDM course used the OPTION framework. **Mandatory course.**	The CoMeD OSCE exam comprised four scenarios including one about SDM. Authors did not publish a score on the SDM portion of the OSCE. Students found SDM more challenging than other types of encounters. They were mostly not able to quit a paternalistic conversation technique and unable to evaluate and appreciate the patient's perspective.	Yes (SDM course is now part of psycho-oncology)
**Solomon, 2004**	USA	Quasi-experimental	47 3^rd^ year family medicine clerkship students.	6-hour educational module embedded in internal medicine clerkship teaching participatory decision making (PDM), according to Braddock’s model. PDM is first presented by Faculty, discussed in small groups and practiced using web-based case simulations. **Mandatory course.**	The module was evaluated using a simulated patient experience station where students were given a ‘patient file’ and subsequently met the simulated patient. Students who completed the PDM module were performing marginally better than the students who had not completed the module. However, there was no statistical significance.	Yes
**Towle, 2014**	Canada	Observational	21 first-year medical students.	2 to 3 sessions of 2 hours per semester for three semesters (12 to 18 hours in total). Groups of four students and a patient mentor met to cover topics such as patient-centered care and SDM. **Elective course**	Students' journal entries and responses to a questionnaire indicated that they met program goals and learned the meaning of good communication and collaboration.	Yes
**Wadland, 2011**	USA	Quasi-experimental	277 3rd year medical students.	SDM training integrated into a smoking cessation training program (SDM course lasted 2 hours). Overview of SDM and role plays with colleagues to discuss whether or not patients wished to quit smoking. **Mandatory course.**	Medical students achieved high levels of performance in smoking cessation counseling using the SDM approach. However, there was no statistically significant difference between the performance of the cohort who received the SDM counseling training and the cohort who received regular counseling training.	Yes
**Yedidia, 2003**	USA	Quasi-experimental	293 3rd year medical students from 2 universities.	Duration unclear. Comprehensive communication curricula developed at each school. All curricula shared a standardized teaching approach and promoted core skills: determining the reason for the patient's visit, understanding the patient's perspective, sharing information with the patient, providing education, negotiating, agreeing on a plan, and achieving closure. **Mandatory course.**	Standardized patients assessed student performance in objective structured clinical examinations (OSCEs). On negotiation and shared decision making, students in the intervention group performed better than the students in the control group (mean difference of 5.7%, 95% CI, 4.5–6.9%, p < .001).	No information provided (no response from author)

#### Features of included SDM courses

Eight out of 11 included courses, including all five US-based courses, delivered SDM training during the third year of medical education. One intervention was implemented in the first, second, and fourth year each. Training courses delivered in the third year were usually integrated in clinical clerkship.[26, 27, 31, 33] Nine SDM courses were integrated into an organ-based[24] or symptom-based module,[24, 28, 29] or embedded into a specific training program.[19, 22, 23, 25, 26, 31, 32] Only one SDM training course was standalone.[27] The average reported length of SDM training was 4.2 hours, ranging from one to 18 hours. Only one SDM training course was elective.[30]

Six courses began with introducing a theoretical framework and concepts related to SDM.[22, 24–27, 33] Hoffman et al. used the five step framework for communicating evidence for participatory decision making.[22, 34] Solomon et al. used Braddock’s model for informed decision making[33, 35]. The other four studies did not specify which theoretical framework had been used. Most training courses emphasized the integration of SDM skills in clinical training and incorporated an experiential approach. In Canada, a unique pilot program involved patients as health mentors who assumed the role of teacher for a group of students over three semesters.[30] Three additional studies utilized standardized patients.[31–33] Several courses included role plays.[22–27] Small group learning approaches to facilitate discussion and generate feedback were also common, ranging from four to 18 students per small group.[22–25, 27–30]

### Impact on SDM skills

Nine studies assessed students’ skills in SDM and communication in healthcare. Three studies quantitatively evaluated students using the objective structured clinical examinations (OSCE), which assess clinical skills including patient communication.[24, 28, 29, 32] Yedidia et al. concluded that a dedicated communication curriculum demonstrated an improvement in overall communication competence.[32] The intervention group scored significantly higher in negotiation and SDM compared to the control group (5.7% difference, p < .001). Kiessling et al. noted that third year students’ communication skills were assessed using OSCE, but did not report the scores. Mortsiefer et al. reported that between 10.2% and 11.3% of students failed the OSCE but did not provide specific information or scores on SDM.

Towle et al. took a qualitative approach to evaluate students’ SDM skills. The authors analyzed students’ journal entries and performed a thematic analysis of questionnaire responses to assess SDM skills. The authors concluded that students were learning the practical meaning of good communication and collaboration.[30]

Two studies used a pre-post approach to determine changes in students’ SDM skills.[22, 26] Using a pre-post control group design, Marko et al. demonstrated a significant improvement in students’ ability to communicate with patients using SDM to deliver bad news and explain treatment options. Their improvement was significantly higher compared to the control group (94.2% vs 69.2%, p < .001). Hoffmann et al. used the Observing Patient Involvement (OPTION) scale and Assessing Communication about Evidence and Patient Preferences (ACEPP) tool to rate recorded role-plays and measure SDM skills. After the training, the intervention group scored significantly higher on the OPTION scale (19% improvement: 30.9 at baseline and 49.1 post-intervention, p < .001) and the ACEPP items (18% improvement: 3.2 at baseline and 4.1 post-intervention, p < .001).

Four studies developed their own measures to assess the course’s impact on SDM skills.[23, 27, 31, 33] Morrow et al. recognized the challenge in assessing students’ SDM skills using existing measures of communication as communication skills do not correlate well with SDM skills. To provide individual feedback to students, the authors created a tool based on the Ottawa Personal Decision Guide. The authors did not report the results of the evaluation. Wadland et al. had faculty evaluate students’ videotaped performances in clinical scenarios using a 30-point scale on SDM and smoking cessation. There was no significant difference on students’ overall performance between the cohort that included SDM approach and the one that did not. Hulsman et al. developed the Academic Medical Centre Communication Test (ACT), presenting films and corresponding essay questions. The students who passed the exam scored relatively poorly on decision making compared to history taking (t = 5.0; d.f. = 109; p < .001) and breaking bad news (t = 3.5; d.f. = 109; p < .001), which may be a result of the relatively few sessions dedicated to decision making within the communication training. Solomon et al. determined students’ SDM skills using simulated patients and a rating scale with 11 questions. Students who completed the participatory decision making module performed better than the students who had not completed the module. The difference, however, was not statistically significant (p < .05).

### Impact on confidence practicing SDM

Three studies examined students’ confidence practicing SDM.[22, 24, 27] As there is no suitable measure for evaluating confidence, Hofmann et al. developed a 10-item, 100-point scale to evaluate confidence in facilitating SDM where scores were significantly better in the intervention group (74.1 at baseline, 87.8 post-intervention, p < .001).

Kiessling et al. evaluated students’ confidence in communication skills using a pre-post design. Students responded to items related to topics covered in the course on a scale of 1 to 5, (1 = totally secure and 5 = totally insecure). They observed that students were more confident in all aspects of communication, including dealing with patients’ emotions (3.3 vs 2.6, p≤ .001), sharing complex information (2.8 vs 2.2 p≤ .001), gathering the relevant information from patients (2.6 vs 2.0, p≤ mail to .001), and understanding what the patient is telling (2.8 vs 2.3, p = .003). Students enrolled in Morrow et al.’s study gained confidence in using SDM and reported that the SDM curriculum helped them feel “more confident and competent in engaging a patient in a SDM… to help the patient come to a decision best suited for them.”

### Impact on attitudes towards SDM

Five studies examined the impact of the training course on students’ attitudes towards SDM.[22, 24, 25, 28, 30] In Australia, a randomized controlled trial of a one-hour SDM training effectively improved students’ attitudes towards patient-centered communication as measured using the Patient Practitioner Orientation Scale (PPOS).[22] The intervention group scored significantly higher on the sharing sub-scale, demonstrating their belief that patients should be informed and included in the decision-making process.[22]

Kiessling et al. found that many students’ personal goals aligned with the course objectives, including gaining more confidence in working with patients and their emotions. Students in Towle et al.’s study reported satisfaction with the course and intention to use the skills in practice. Luttenberger et al. reported that the majority of the students found the course useful for their job as a doctor.

Mortsiefer et al. documented challenges with the course. Students had a difficult time changing their communication style from a paternalistic approach to appreciating and evaluating patients’ perspectives. The lack of role models and instruction in SDM were mentioned as potential barriers, and the students approved curriculum reforms to include training in SDM. The Communication in Medical Education Dusseldorf (CoMeD) course received some of the highest ratings from students. Many students who took CoMeD reported that they planned to use the newly learned skills in their practice and found them to be applicable across different specialties.[28]

Morrow et al. gathered and identified themes from students’ feedback. One of the themes indicated that understanding of SDM concepts improved. Students found it helpful to review patients’ preferences and saw the benefits of SDM for the patients: “You can never go wrong with SDM—you leave with a certain confidence that the patient understands the choices he/she made, rather than the ones you impose.”[27]

### Routine integration in the medical curriculum

Ten training courses continue to be part of medical education.[22–31] Hoffmann et al. described the SDM training’s design as intentionally brief to promote integration into existing curricula.[22] Several studies noted the importance of the teachers’ roles and related training and commitment. In Germany, Luttenberger et al. concluded that, with practical training, it is feasible to implement SDM training courses without additional time or staff.[25] They found that the only required resource is the teacher and recommended one staff member per 20 students. According to Mortsiefer et al., embedding communication teaching in a clinical context and involving clinicians as lecturers are important in ensuring relevance and achieving acceptance by students. They considered communication strategies to be directly linked to students’ understanding of the disease.

## Discussion and conclusion

### Discussion

This review is the first to examine the impact of SDM training courses embedded in undergraduate medical school curricula. Most SDM courses were delivered in the third year of school and emphasized an experiential approach to integrate SDM skills into clinical training. All but one were compulsory and embedded in other modules or programs. Course length varied from one to seven and a half hours. Most studies suggested that medical students’ skills in SDM significantly increased post-training. Students also reported more confidence in promoting SDM. All studies that measured the impact on students’ attitudes (n = 5) reported a positive effect of the SDM course. Three studies reported no significant improvement in SDM skills. Ten courses continue to be taught routinely. Study authors suggested that the integration of SDM training courses in undergraduate medical curricula was feasible and did not require additional resources. This seemed highly dependent on the lecturer’s skills and interest in SDM. Prioritizing clinician lecturers was considered important. Given the variation in duration, format, clinical context, and assessment methods, we are unable to determine which training format is most effective. It is worth noting that only one SDM training course was standalone.

#### Results in context

An environmental scan of SDM training programs for health professionals found similar variation in the teaching methods, duration, evaluation methods, and evidence of impact.[17] Consistent with the present review, there was no evidence to indicate which training method was most effective and no comparison between theoretical and experiential training. Most studies included in this review combined a theoretical approach and experiential session. Consistent with Legare et al.’s findings, we found no evidence of SDM training for undergraduate medical students in middle- and low-income countries. Contrary to Legare’s environmental scan, none of the studies included in this review evaluated the course’s impact on knowledge of SDM.

#### Strengths and limitations

The main strength of this review is the use of a five-stage framework that maximizes transparency and reproducibility. Other strengths include the involvement of two researchers to independently screen, select, and extract data, as well as the inclusion of a consultation exercise as recommended by Arksey and O’Malley. A limitation of this study is the exclusion of articles not published in English.

## Conclusion

Despite the heterogeneity of included studies, the scoping review findings indicated that nine out of 11 training programs were effective in improving undergraduate medical students’ skills, confidence, or attitudes regarding SDM. They also appeared feasible to implement. None of those studies undertook follow-up assessments beyond two weeks and therefore cannot demonstrate whether skills taught during undergraduate medical education would influence students’ behaviors as attending clinicians. Further research is needed. Some authors have advanced that because of the complex communication and clinical skills involved, SDM should be taught once health professionals have developed adequate technical experience.[36] The findings of this review suggest otherwise, indicating that undergraduate medical training appears feasible and likely to be effective, at least in the short term, as a valuable introduction to the importance of listening to patients’ voices and facilitating preference construction. Importantly, 10 out of 11 programs continue to be taught routinely, thus suggesting that embedding SDM training in undergraduate medical education is feasible and may be a potential solution to current implementation challenges.

### Practice implications

Shared decision making training has shown to effectively improve patients’ experiences with care and some outcomes related to cognition.[5] While SDM instruction is increasingly embedded in continuing medical education and has received burgeoning support in health policy worldwide, little emphasis has been placed on integrating its instruction in undergraduate medical school curricula. Overall, the studies presented in this scoping review indicate the feasibility, acceptability, and potential positive, short-term implications of embedding SDM instruction in undergraduate medical education. Further, instructing undergraduate medical students in SDM may present an effective solution to implementation barriers, especially those related to stigma and misunderstanding. Ultimately, SDM empowers patients to become more actively involved in and have a better understanding of their healthcare, and instructing future physicians in its principles and use may stimulate uptake in clinical settings.
